# Simultaneous analysis of two drugs used as supportive treatment for COVID-19: comparative statistical studies and analytical ecological appraisal

**DOI:** 10.1186/s13065-022-00860-8

**Published:** 2022-09-27

**Authors:** Hany Ibrahim, Omar M El-Abassy, Hisham Ezzat Abdellatef, Hassan A. M. Hendawy, Heba M El-Sayed

**Affiliations:** 1 Pharmaceutical Chemistry Department, Faculty of Pharmacy, Egyptian Russian University, Badr City, Cairo, 11829 Egypt; 2 Department of Analytical Chemistry, Faculty of Pharmacy, Zagazig University, Zagazig, 44519 Egypt; 3 Pharmaceutical Chemistry Department, Egyptian Drug Authority, Giza, Egypt

**Keywords:** COVID-19, Ratio subtraction, Induced dual wavelength, Fourier self-deconvolution, Ecological appraisal

## Abstract

Pharmaceutical quality control products (QC) demand quick, sensitive, and cost-effective methods to ensure high production at a low cost. Green analytical methods are also becoming more common in pharmaceutical research to cut down on the amount of waste that goes into the environment. Meclizine hydrochloride (MZH) and pyridoxine hydrochloride (PYH) are reported to be excellent for calming down COVID-19. As a result, the amount of MZH and PYH manufactured by multinational pharmaceutical organizations has increased considerably during the last several months. The present work proposes three environmentally friendly, straightforward, and sensitive spectrophotometric procedures for quantification of MZH in the presence of PYH in a pure and marketable formulations. The approaches under examination include ratio subtraction (RSM), induced dual wavelength (IDW), and Fourier self-deconvolution (FSD). PYH, on the other hand, was directly quantified at 290 nm. For both drugs, the procedures follow Beer’s law in the range of (5–50 µg/mL). The RSM, IDW, and FSD methods, as well as the zero-order approach for PYH, have all been verified in accordance with ICH standards. The ecological value of established methodologies was determined using four distinct ways: the national environmental methods index (NEMI), the analytical Eco-scale, the Analytical Greenness Metric (AGREE), and the green analytical process index (GAPI). Comparing the findings to those of the previously described spectrophotometric technique, no major changes were identified.

## Introduction

COVID-19, a novel coronavirus epidemic, was first reported in late December 2019. Since then, it has spread rapidly throughout the world, putting enormous strain on public health systems. As of August 10th, 2021, the World Health Organization (WHO) reported 203,295,170 confirmed cases and 4,303,515 confirmed deaths worldwide. On June, 2021, America had the highest excess mortality rate (six hundred forty thousand), followed by Russia with five hundred thousand by April, 2021, Brazil with five hundred thousand by May, 2021, and Mexico with four hundred seventy thousand by May, 2021 [1]. A number of medications, including MZH and PYH, are available may aid in the management of COVID-19. Treatment with a combination of chloroquine, corticosterone, meclizine, and pyridoxine may be effective in preventing blood clotting, which in turn may help to keep the infection from spreading. Thus, drugs such as chloroquine, cortisol, meclizine, and pyridoxine may be used to prevent and cure coronavirus infection, as well as to treat them after they have occurred [2].

**Fig. 1:**
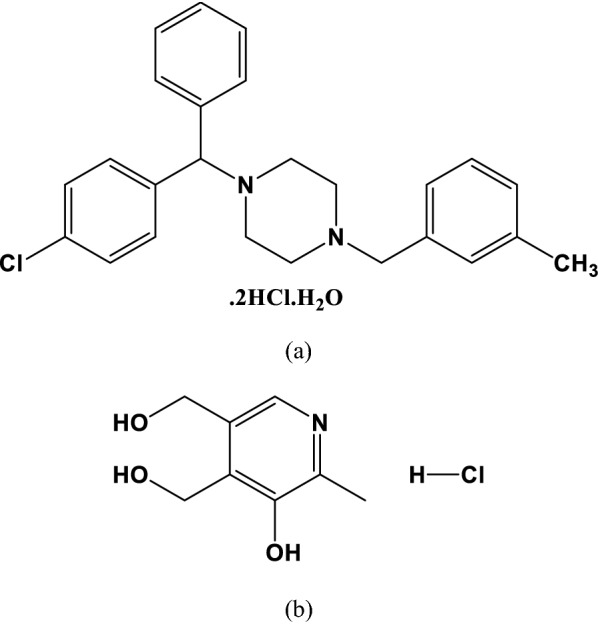
Chemical structure of **a** meclizine hydrochloride and **b** pyridoxine hydrochloride

Meclizine hydrochloride (MZH) (1-(4-chlorobenzhydryl)-4-(3-methylbenzyl) piperazine dihydrochloride), Fig. 1a, is a sedating antihistamine having antimuscarinic and sedative properties. It is most recognized for its antiemetic qualities, which have been reported to last up to 24 h. MZH is used to treat vertigo caused by Meniere's illness and other vestibular diseases, as well as to prevent and cure nausea, vomiting, and inflammation induced by COVID-19 alone or in combination with pyridoxine [3–6]. Pyridoxine hydrochloride (PYH), popularly known as vitamin B6, is (5-hydroxy-6-methylpyridine-3, 4-diyl) dimethanol hydrochloride Fig. 1b. When combined with an antihistaminic, it is the first-line therapy for vomiting and nausea during pregnancy [7]. Pyridoxine’s anti-oxidative and anti-inflammatory capabilities may have a therapeutic effect in reducing the intensity of COVID-19 and its effects [8]. MZH has been determined with PYH using a variety of analytical methods, including spectrophotometry [9–15], HPLC [14, 16–20], and UV and/or chemometric approaches [9], according to inquiry of the literature.

Although UHPLC and HPLC as analytical instruments have a high degree of sensitivity and selectivity, they are more sophisticated and require a greater investment in equipment maintenance and analysis time. Additionally, before injection, the sample must be cleaned. Although UV-Spectrophotometry is a rapid, sensitive, and inexpensive technique for analysis, it is difficult to apply direct UV-Spectrophotometric techniques to the examination of binary pharmaceutical formulations, owing to spectrum overlap and a lack of specificity. Today, new spectrophotometric techniques that use simple software and math are used to separate overlapping spectra [21].

It became common to use green analytical chemistry (GAC) in the early 2000s [22]. Researchers in this emerging field are working to reduce the use of hazardous chemicals in traditional analytical procedures while also improving analyst and environmental safety [23]. Procedural safeguards have recently been established to minimize or restrict the potentially hazardous impacts of analytical procedures. Reusing, replacing with greener alternatives, and cutting back on the use and decontamination of reagents and solvents are some of the main solutions.

Researchers in this work used a simple and sensitive UV-Spectrophotometric approach to measure both MZH and PYH in pure and different marketable formulations at the same time, using three novel approaches for the first time. No reported method describes analysis of MZH and PYH using RSM, IDW and FSD. There were four standard techniques for assessing the suggested approach ecologically, and it was shown to be more ecologically friendly in each step. Statistical comparisons have been done to show that there is no significant difference in the results of the proposed method with reported methods and with each other using six statistical comparison tools. Peak resolution was also based on zero-order spectra of analyte, so complex software or mathematical manipulation was not required. As a result, the suggested strategy has been rigorously validated in accordance with ICH criteria [24], confirming its reliability in everyday use. Aside from being ecologically friendly, this procedure is also simple and cost-effective.

## Experimental

### Instruments and software

A double beam spectrophotometer (Jasco, Japan) was used for all spectrophotometric measurements. Spectrum treatment was achieved using Jasco spectra manager software. In pharmaceutical sample preparation, a sonicator (DAIHAN WUC-A01H, USA) has been used. Minitab 2019 was used in the statistical comparison survey for the results obtained and reported.

### Materials and reagents

MZH and PYH pure standards were kindly supplied by EIPICO (Tenth of Ramadan City, Egypt). The purity was found to be 99.5 and 99.8 percent for MZH and PYH, respectively, according to the analysis certificate of the manufacturer. HPLC grade ethanol was received from Thermo fisherman (USA).

### Pharmaceutical formulation

Navoproxin plus^®^ tablets, Batch number BN25323, Vomidoxine B6^®^ tablets BN1360002 and Dizirest B6^®^ tablets BN10918 were purchased from local market. They were labelled to contain 25 mg MZH and 50 mg PYH per tablet and manufactured by Delta Pharmaceutical Industries, Sigma Pharmaceutical Industries, and Pharaonia Pharmaceuticals, respectively.

### Standard stock solution

MZH and PYH (100 µg/mL) standard stock solutions were prepared by weighing and precisely transferring 10 mg of every single standard powder into a 100 mL volumetric flask. After that, they were dissolved and sonicated into 70 mL ethanol for 15 min, and the volume was completed to 100 mL using ethanol.

### Construction of calibration curves

PYH at final concentrations of (5–50 µg/mL) was scanned in the wavelength range of 200–400 nm against ethanol as a blank, and the absorbance at 290 nm was measured directly without interference from MZH. The regression equation was derived by constructing a calibration curve relating the absorbance at 290 nm to the relevant PYH concentrations, while techniques for MZH include the following methods.

### RSM

The zero order absorption spectrum of the prepared solutions (5–50 μg/mL) were scanned at 200–400 nm against ethanol as a blank. The absorbances of MZH working solutions were computed at 230 nm after dividing by spectra of 30 µg/mL PYH, then subtracting the constant value of the plateau region, followed by multiplication of the obtained spectra by the divisor (30 µg/mL PYH). Calibration curve of MZH was then fabricated relating the obtained absorbances at 230 nm and the corresponding drug concentrations.

### IDW

MZH serial dilutions in the range of (5–50 µg/mL) were scanned and the corresponding zero order absorption spectra were recorded. The amplitude differences at 230 and 245 nm were plotted against their respective concentrations, and an equality factor (F_eq_) was calculated for PYH where 245 nm had amplitude values multiplied by the F_eq_.

### FSD

The recorded zero-order spectra for the tested medications were deconvoluted by Fourier wavelet function, using 55 as the full width at half maximum value (FWHM). The produced amplitudes of MZH at 226 nm were then plotted against their respective concentrations (5–50 µg/mL).

### Analysis of laboratory mixtures

Various laboratory mixtures in different complementary ratios (5:10, 6:12, 15:15, 40:20, and 50:25, MZH: PYH in µg/mL) were prepared using stock solutions of analytes to investigate several analytical and validation considerations of the proposed methods. Each recommended method regression equation was then used to quantify each component in the laboratory prepared mixes.

### Analysis of pharmaceutical formulations

Ten tablets of each studied drugs were weighed and finely crushed. An amount equivalent to one tablet was precisely weighed and placed in a 100-mL volumetric flask, then ultrasonicated with 50 mL ethanol for 15 min. After cooling, the solution was diluted to volume with ethanol and filtered to achieve a stock solution containing 250 µg/mL MZH and 500 µg/mL PYH. The stock solution was further diluted with ethanol to obtain different concentrations of MZH and PYH within linearity range. The prepared samples were measured according to the procedure described under the construction of MZH and PYH calibration curves. Each drug concentration was estimated from the corresponding regression equation.

## Results and discussion

**Fig. 2:**
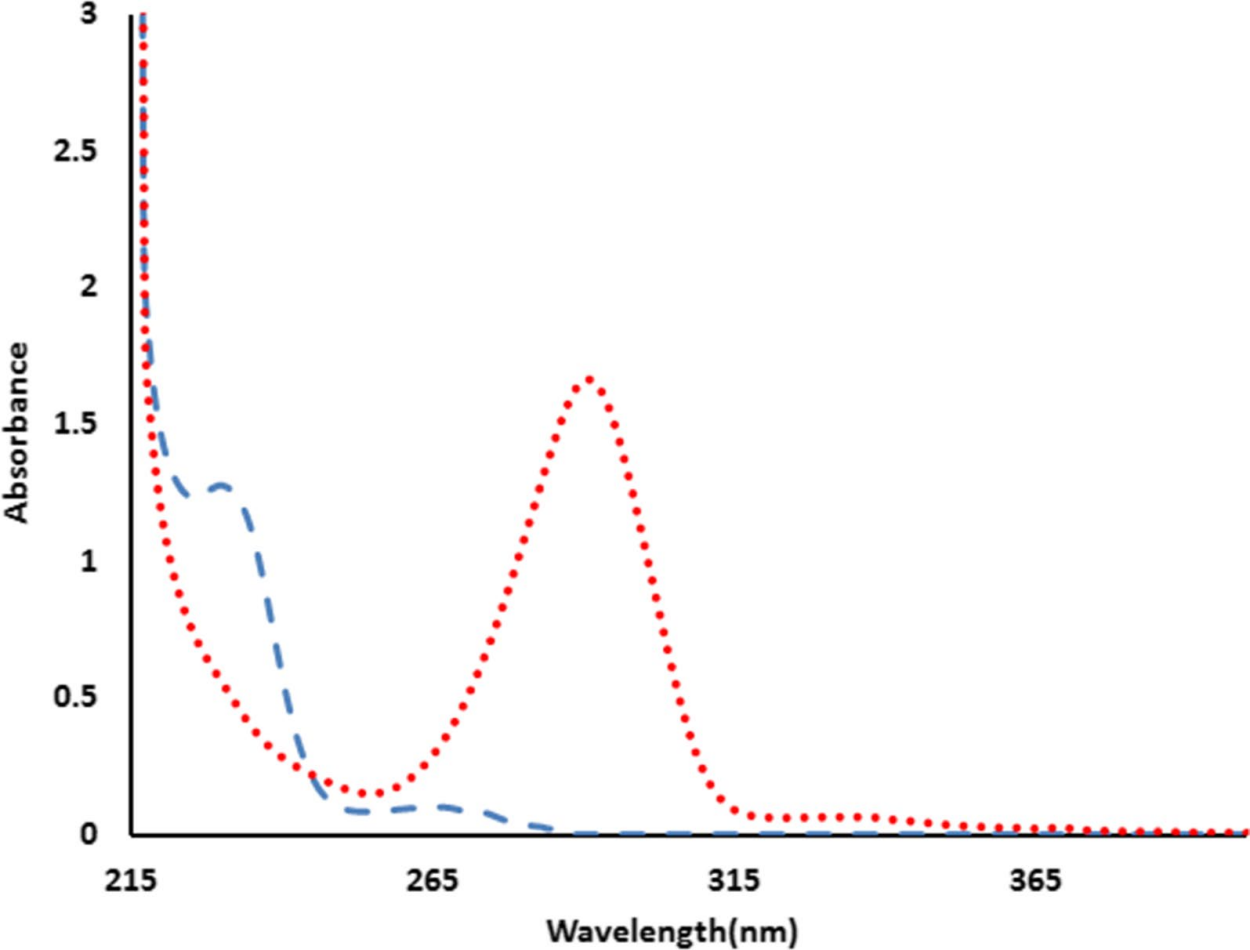
Zero-order absorption spectra of 35 µg/mL of meclizine hydrochloride (blue dashed line), 35 µg/mL of pyridoxine hydrochloride (red dotted line) in ethanol

UV scanning of a mixture containing MZH and PYH shows sever overlapped spectra (Fig. 2). Therefore, three unique, time-saving, cost-effective, sensitive and simple UV-spectrophotometric platforms were introduced for selective analysis of MZH by eliminating interference of PYH. The following developed methods were used for the quantitation of MZH and PYH simultaneously in their synthetic binary mixtures and pharmaceutical preparations.

### RSM

**Fig. 3:**
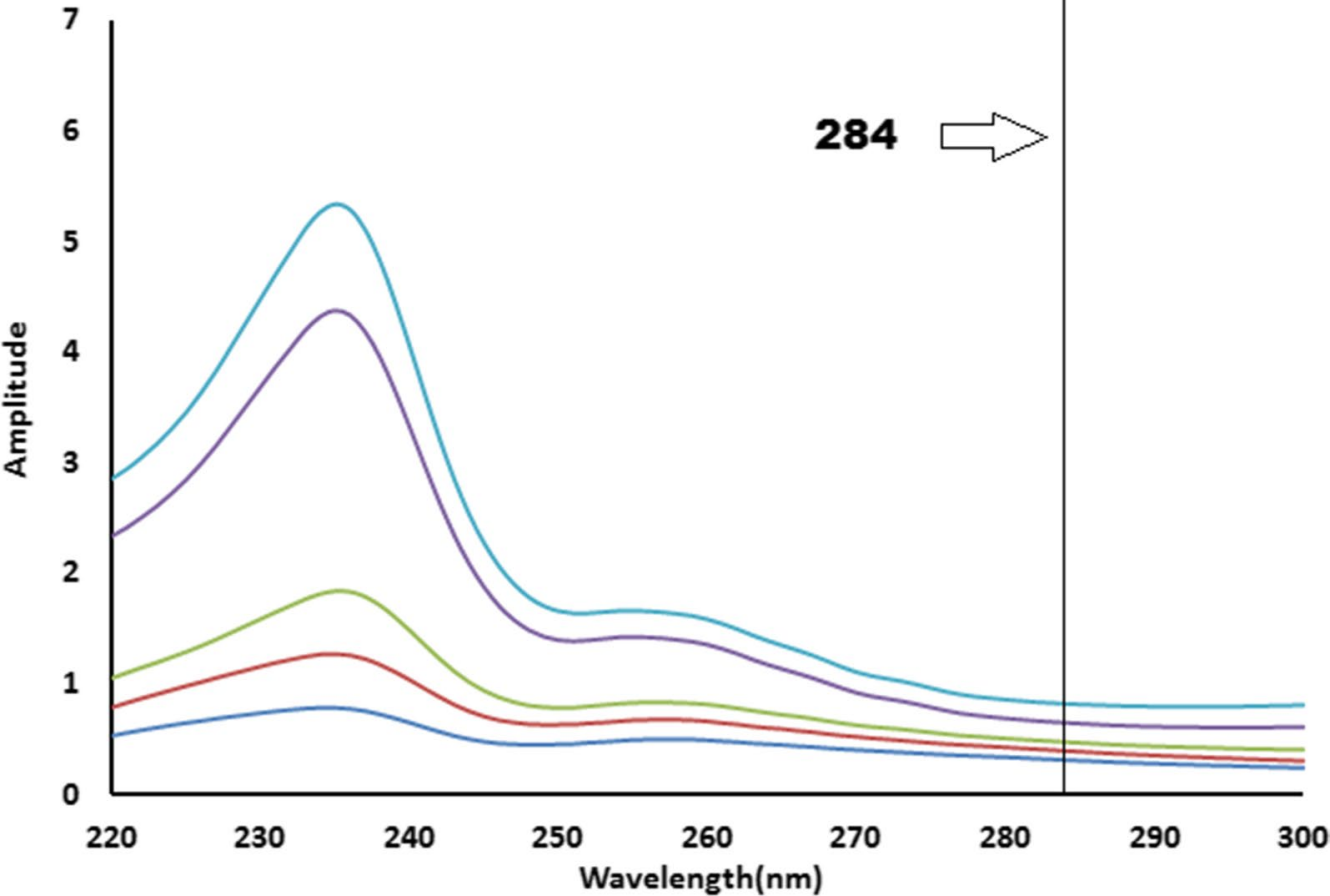
Ratio spectra of different laboratory prepared mixtures of meclizine hydrochloride and pyridoxine hydrochloride using 30 μg/mL of pyridoxine hydrochloride as divisor and ethanol as a blank

**Fig. 4:**
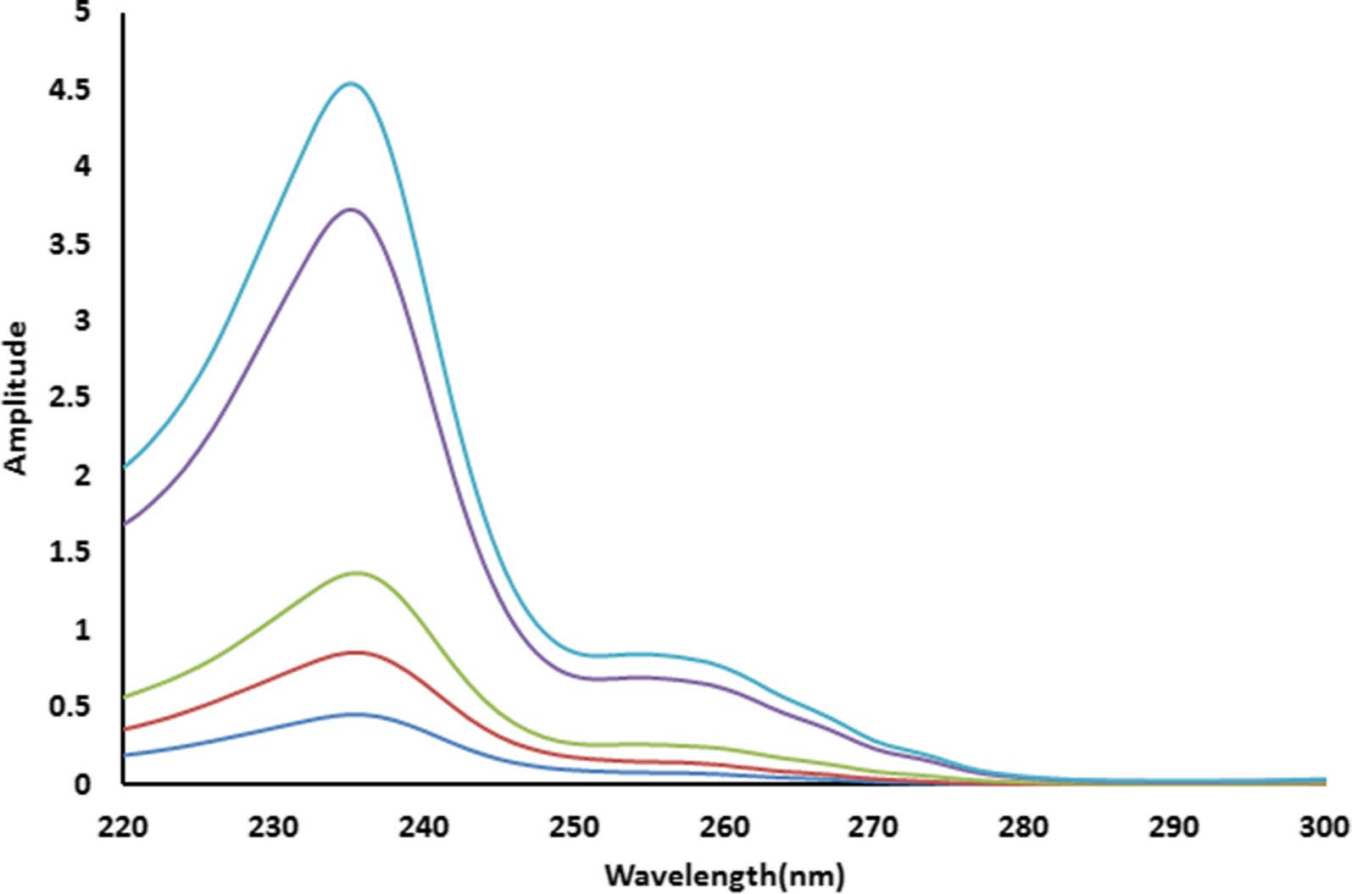
Ratio spectra of laboratory prepared mixtures of meclizine hydrochloride and pyridoxine hydrochloride using 30 µg/mL of pyridoxine hydrochloride as a divisor and ethanol as a blank after subtraction of the constant

**Fig. 5:**
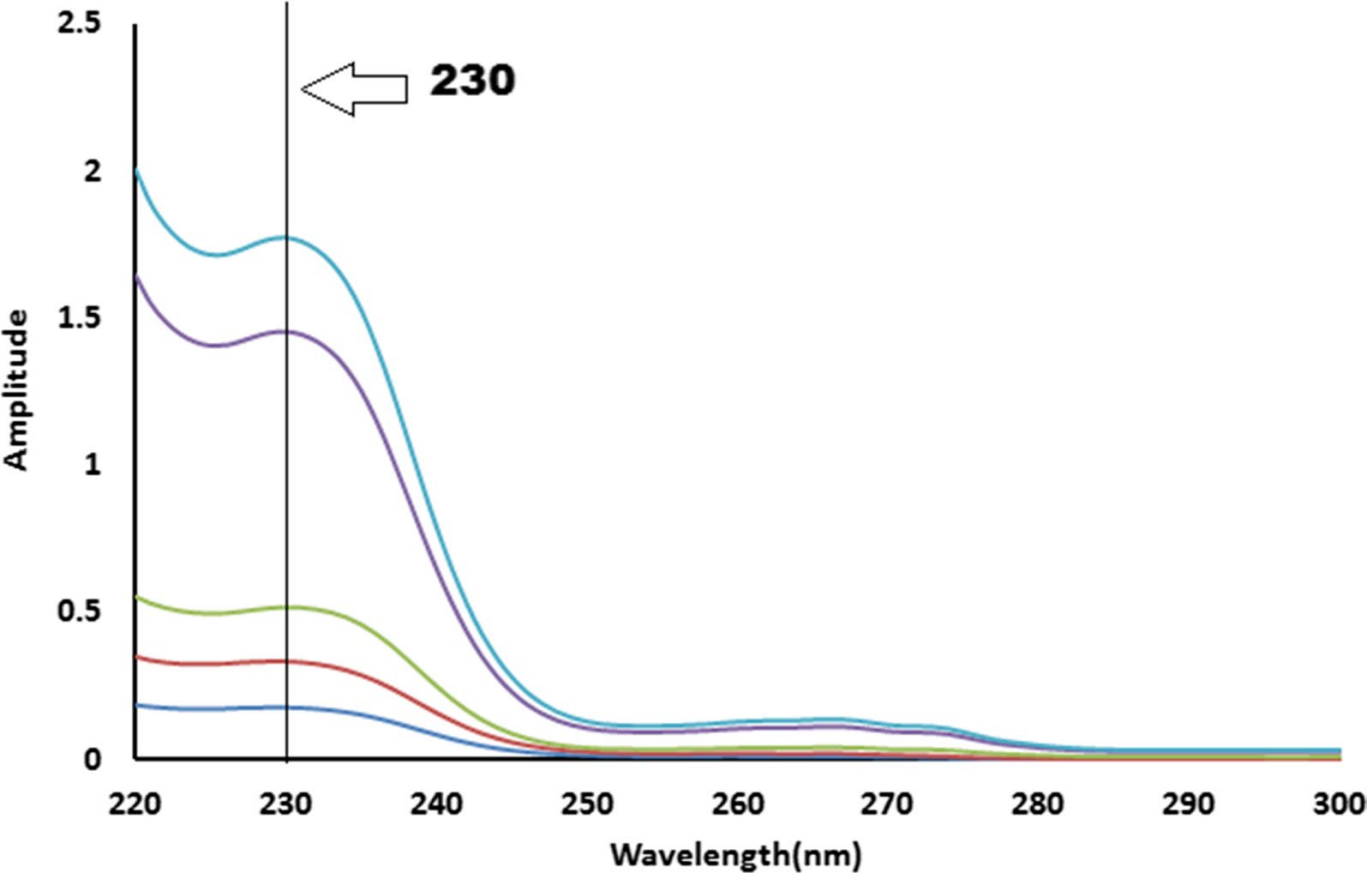
The zero order absorption spectra of meclizine hydrochloride obtained by the proposed ratio subtraction method for the analysis of laboratory prepared mixtures after multiplication by the divisor

The RSM method [25] was employed to resolve the overlapped spectra of MZH and PYH (Fig. 2) by scanning the zero order absorption spectra of the laboratory-prepared mixtures (MZH and PYH), dividing them by a cautiously chosen concentration of standard PYH (30 µg/mL) as a divisor. The produced ratio spectra represent MZH/PYH+ constant as shown in Fig. 3, then subtracting the values of these constants PYH/PYH in the plateau region (284–300 nm) as presented in Fig. 4, followed by multiplication of the acquired spectra by the divisor PYH (30 µg/mL) as presented in Fig. 5.

### IDW

IDW approach is based on cancelling the absorbance of an interfering element in the zero order overlapped spectra by calculating F_eq_ (the ratio between absorbance values for the interfering analyte at two specified wavelengths; A λ_1_/A λ_2_) [26]. The absorbance difference (∆A) for the component in interest was calculated after multiplication of its absorbance at λ_2_ by F_eq_ then correlating ∆A to the corresponding concentration. In this study, the absorbance of PYH was equalized by assessing F_eq_ (A 230/A 245), while ∆A of MZH at the selected wavelengths was high. MZH absorbance at 245 nm was multiplied by F_eq_, then ∆A was calculated, and the regression equation was used for back calculation of MZH concentration. The λ_max_ of MZH (230 nm) was chosen as one of the two wavelengths to increase the magnitude of ∆A values and enhance method sensitivity.

### FSD

**Fig. 6:**
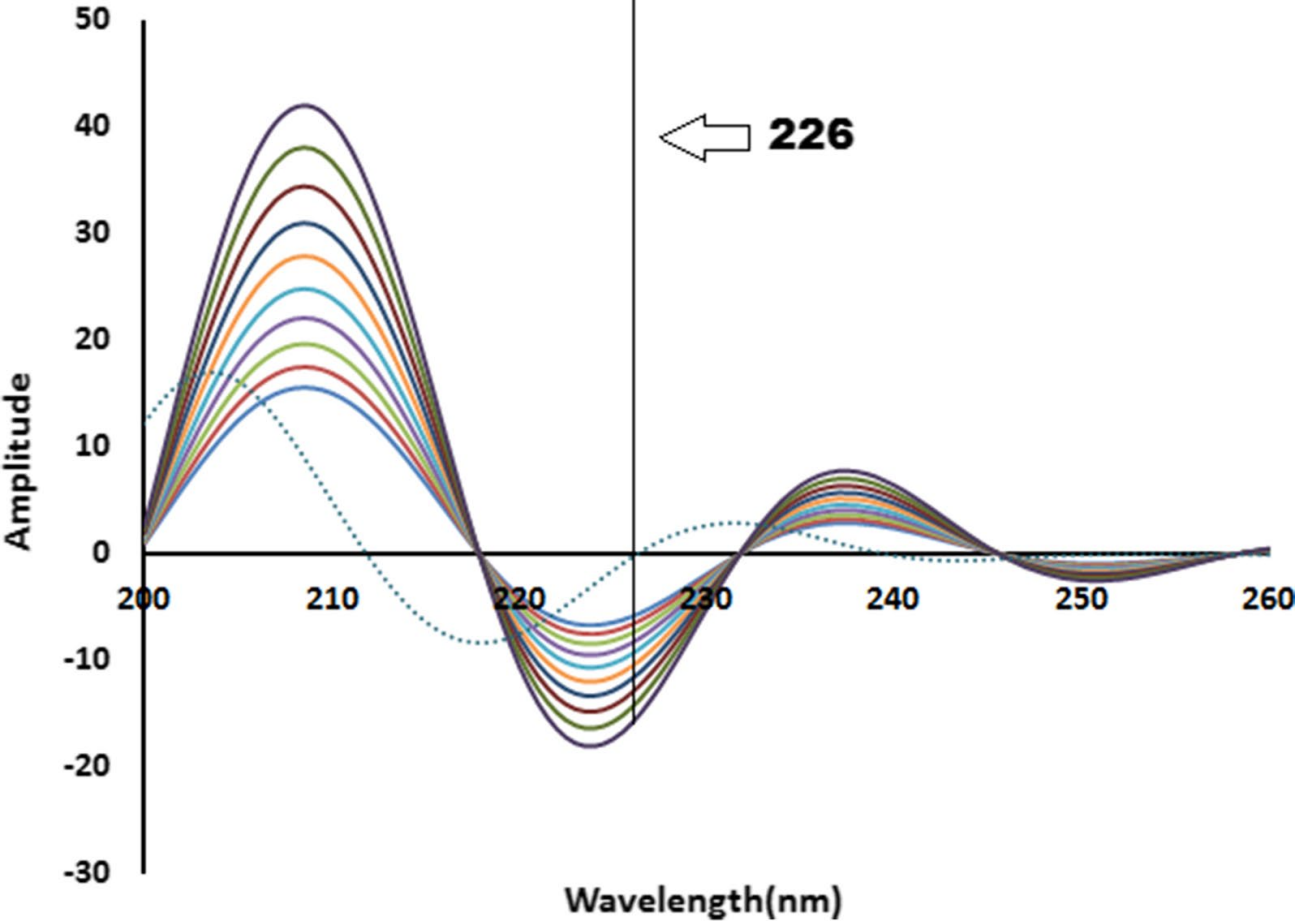
Deconvoluted spectra of 5–50 µg/mL of meclizine hydrochloride (solid line) computed at 226 nm where zero-crossing point of pyridoxine hydrochloride deconvoluted spectrum (blue dotted line)

FSD method is a novel spectrophotometric method used for analyzing binary mixtures [27–29]. It is a simple straightforward mathematical technique for resolving severely overlapped zero-order spectra by compressing their bandwidth using Fourier or deconvolution feature of spectrophotometer software [27]. Zero-crossing or no-contribution sites were obtained by overlaying the medicinal combinations spectra and allowed determination of one component without influence from the other. MZH concentration was back calculated from regression equation relating the amplitude of deconvoluted MZH spectrum was recorded at 226 nm and the relevant concentration ranges of 5–50 g/mL, Fig. 6.

### Method validation

Linearity, the limit of detection (LOD), the limit of quantitation (LOQ), selectivity, accuracy, and precision were all tested according to the ICH Q2 (R1) criteria [24].

### Linearity

Linearity of the proposed spectrophotometric methods for MZH and PYH quantitation was tested by measuring different concentration absorbances in the ranges provided in Table 1 in triplicates. The adopted methods exhibited good linearity (correlation coefficient, R ≥ 0.9995). Table 1 shows the regression parameters of the proposed methods.

**Table 1:** Validation data for determination of MZH and PYH by the proposed methods

Validation parameters	MZH			PYH
	RSM	IDW	FSD	Direct
Wavelength (nm)	230	230 and 245	226	290
Linearity (µg/mL)	5–50	5–50	5–50	5–50
Slope	0.03	0.02	0.22	0.04
Intercept	− 0.02	− 0.01	4.12	− 0.04
Correlation coefficient (R)	0.9998	0.9997	0.9995	0.9999
LOD (µg/mL)	0.52	0.87	1.11	0.43
LOQ (µg/mL)	1.60	2.65	3.37	1.31
Accuracy (recovery% ± SD)^a^	100.32 ± 0.86	99.40 ± 1.63	99.07 ± 1.11	100.46 ± 1.22
Precision (% RSD)				
Intra-day	0.97	0.54	0.81	0.77
Inter-day	1.20	0.86	1.03	0.93

^a^Mean of five determinations

### The limits of detection (LOD) and limits quantification (LOQ)

According to ICH Q2 (R1) recommendations, the LOD and LOQ were obtained by determining the lowest concentrations that could be detected and quantitatively measured, respectively, as indicated in Table 1.

$${\text{LOD}} = 3.3\;{\text{S/b}}\;{\text{and}}\;{\text{LOQ}} = 10\;{\text{S/b}}$$

where S is the standard deviation of the intercept of the calibration curve, and b is the slope of the calibration curve.

### Accuracy

The suggested methods accuracy were assessed by comparing five acquired concentrations of each drug to their real values. The calculated mean percentage recoveries are presented in Table 1.

### Precision

The precision of each proposed techniques was tested intraday by repeating the determination of 10, 25, and 40 µg/mL of each analyte three times on the same day. The inter-day precision was evaluated by performing the analysis three times in a row, with the findings reported as RSD in Table 1.

### Selectivity

The proposed methods selectivity were tested by assessing laboratory-prepared mixtures comprising varied MZH: PYH ratios. As indicated in Table 2, the mean recovery percentages were within the acceptable limit.

**Table 2:** Analysis of laboratory prepared mixtures by the proposed methods

Methods	RSM	IDW	FSD	Direct
Concentration (µg/mL)	Found %^a^			Found %^a^
	MZH			PYH
MZH:PYH				
5:10^b^	99.22	98.13	98.99	99.33
6:12^b^	100.37	98.70	100.73	101.54
15:15	101.21	99.33	99.41	98.43
40:20	99.39	100.12	98.09	100.78
50:25	100.84	100.70	99.75	100.04
Mean ± SD	100.21 ± 0.87	99.40 ± 1.03	99.40 ± 0.97	100.02 ± 1.21

^a^Average of five determinations

^b^The same concentration ratio in (Navoproxin plus^®^), Vomidoxine B6^®^, Dizirest B6^®^)

### Analysis of dosage form

The stated spectrophotometric techniques were used to reveal the concentrations of both MZH and PYH in their combined pharmaceutical formulations (Navoproxin plus tablet^®^, Vomidoxine B6^®^ tablets, Dizirest B6 tablets). The validity of the recommended procedures was further evaluated using the standard addition technique, which revealed no interference from excipients. Results of the described procedures exhibited high percentage recoveries as summarized in Table 3.

**Table 3:** Determination of MZH and PYH in different pharmaceutical formulations using the proposed methods using standard addition technique

Drugs	MZH			PYH
Methods	RSM	IDW	FSD	Direct
Navoproxin plus^®^ tablets^a^ (found% ± SD)	99.73 ± 0.88	99.58 ± 1.07	99.06 ± 0.83	100.14 ± 0.69
Navoproxin plus ^®^ tablets^a^ standard addition (recovery% ± SD)^b^	100.18 ± 0.86	99.55 ± 0.78	99.70 ± 0.52	99.54 ± 0.93
Vomidoxine B6^®^ tablets^a^ (found% ± SD)	100.09 ± 0.73	99.87 ± 1.22	99.15 ± 1.27	100.63 ± 0.61
Vomidoxine B6® tablets^a^ standard addition (recovery% ± SD)^b^	100.50 ± 1.08	99.36 ± 0.78	98.65 ± 0.41	99.53 ± 1.72
Dizirest B6^®^ tablets^a^ (found% ± SD)	99.91 ± 1.01	99.66 ± 1.03	99.51 ± 0.90	101.06 ± 0.79
Dizirest B6^®^ tablets^a^ standard addition (recovery% ± SD)^b^	99.19 ± 0.77	99.48 ± 1.22	100.13 ± 0.86	100.13 ± 1.05

^a^Navoproxin plus, Vomidoxine B6 and Dizirest B6 claimed to contain 25 mg/mL of MZH and 50 mg/mL for PYH

^b^Average of five determinations

### Assessment of the proposed approaches environmental impact

It is critical to substitute harmful solvents and reagents with less toxic alternatives if the analytical process is to be environmentally benign. NEMI [30], analytical Eco-scale [31, 32], AGREE [33] and GAPI [32] are all well-known analytical instruments in this sector. The four different assessment tools mentioned above assessed greenness of the analytical methods (Table 4). NEMI is a four-quartered graph. The four green quarts signposted that the solvents used are not dangerous, chronically bio-accumulative, poisonous, or corrosive, and produce insignificant amounts of wastes. Eco-scale is another evaluation system built on penalty points. The procedure starts with a score of 100. The penalty points are subtracted from the base value if it deviates from the ideal. An eco-scale score of 95 was obtained for the recommended approaches. GAPI is a new aspect with five pentagrams representing the environmental effect. The objects are colored green, yellow, or red to indicate low, medium, or major environmental consequences, respectively. AGREE was also recently reported and founded on the twelve principles of GAC. It introduces a clock-shaped graph with twelve pieces around its perimeter, each reflecting a different GAC principle according to its intuitive color and weight reflected by segment width. The color codes for AGREE span from red to yellow to green. The final score and colour in the middle of the proposed methods pictogram confirmed method greenness.

**Table 4:** The outcomes of the evaluation of the proposed approaches greenness

1. NEMI pictogram	2. GAPI	3. AGREE		
				

4. Analytical Eco-scale score			
Item	Number of pictograms	Word sign	Penalty points
Reagents: volume			
Ethanol 10 mL	2	Warning	2
Instrument			
Spectrophotometer			0
Energy [< 0.1 kWh per sample]			0
Waste			3
Occupational hazards (analytical process hermetization)			0
Total penalty points			5
Analytical Eco-Scale score^a^			95 Excellent green method

^a^Analytical Eco-Scale total score = 100 − total penalty points, where score > 75 represents excellent green analysis, score > 50 represents acceptable green analysis, and score < 50 represents inadequate green analysis

### Statistical analysis

The findings of the applied methods were compared to each other and to the results of the reported method [9] using various statistical tools. To compare the suggested and reported approaches, a student t- and F-tests were used, and no significant difference was found, Table 5. The offered and reported approaches were compared using a one-way ANOVA test (Table 6), results revealed that the calculated F-values were less than the critical one, and this indicated no variability between groups, (Table 7) also shows a two-way ANOVA test, which results revealed that no interference from excipients was found in different pharmaceutical formulations.

**Table 5:** Statistical analysis of proposed and reported methods for MZH and PYH in different pharmaceutical formulations

Drug	Method	Navoproxin plus^®^ tablets						Vomidoxine B6^®^ tablets						Dizirest B6^®^ tablets					
		Mean	SD	n	V	t-test^a^	F-value^a^	Mean	SD	n	V	t-test^a^	F-value^a^	Mean	SD	n	V	t-test^a^	F-value^a^
MZH	RSM	99.73	0.88	5	0.78	1.97	2.70	100.05	0.73	5	0.53	1.89	1.61	99.91	1.01	5	1.02	0.24	1.16
	IDW	99.58	1.07	5	1.16	1.43	3.99	99.87	1.22	5	1.48	1.19	1.72	99.66	1.03	5	1.06	0.61	1.12
	FSD	99.06	0.83	5	0.69	0.55	2.39	99.15	1.27	5	1.61	0.14	1.86	99.51	0.90	5	0.81	0.89	1.46
	Reported method [9]	98.81	0.54	5	0.29			99.05	0.93	5	0.86			100.07	1.09	5	1.18		
PYH	Direct	100.14	0.69	5	0.48	0.68	1.84	100.63	0.61	5	0.37	0.81	1.23	101.06	0.79	5	0.62	1.64	1.44
	Reported method [9]	100.50	0.94	5	0.89			100.93	0.55	5	0.30			100.15	0.95	5	0.90		

^a^Theoretical of t (2.306) and F (6.39) values at p = 0.05

**Table 6:** One-way ANOVA results for determination of proposed and reported methods of MZH in different pharmaceutical formulations

Pharmaceutical formulations	Source of variation	Sum of squares	Degree of freedom	Mean of squares	F-value	P-value	Critical F
Navoproxin plus^®^ tablets	Between group	2.78	3	0.92	1.26	0.32	3.23
	Within group	11.75	16	0.73			
	Total	14.53	19				
Vomidoxine B6^®^ tablets	Between group	1.23	3	0.41	0.47	0.70	3.23
	Within group	13.84	16	0.86			
	Total	15.07	19				
Dizirest B6^®^ tablets	Between group	0.92	3	0.30	0.30	0.82	3.23
	Within group	16.43	16	1.02			
	Total	17.35	19

**Table 7:** Two-way ANOVA results for determination of proposed methods of MZH in different pharmaceutical formulations

Source of variation	Degree of freedom	Sum of squares	Mean of squares	F-value	P-value	Critical F
Methods	3	4.54	1.51	1.65	0.18	2.79
Pharmaceutical formulations	2	2.40	1.20	1.31	0.27	3.19
Error	54	49.51	0.916			
Total	59	56.46				

ANOVA was not the only statistical tool utilized to confirm the findings.

**Fig. 7:**
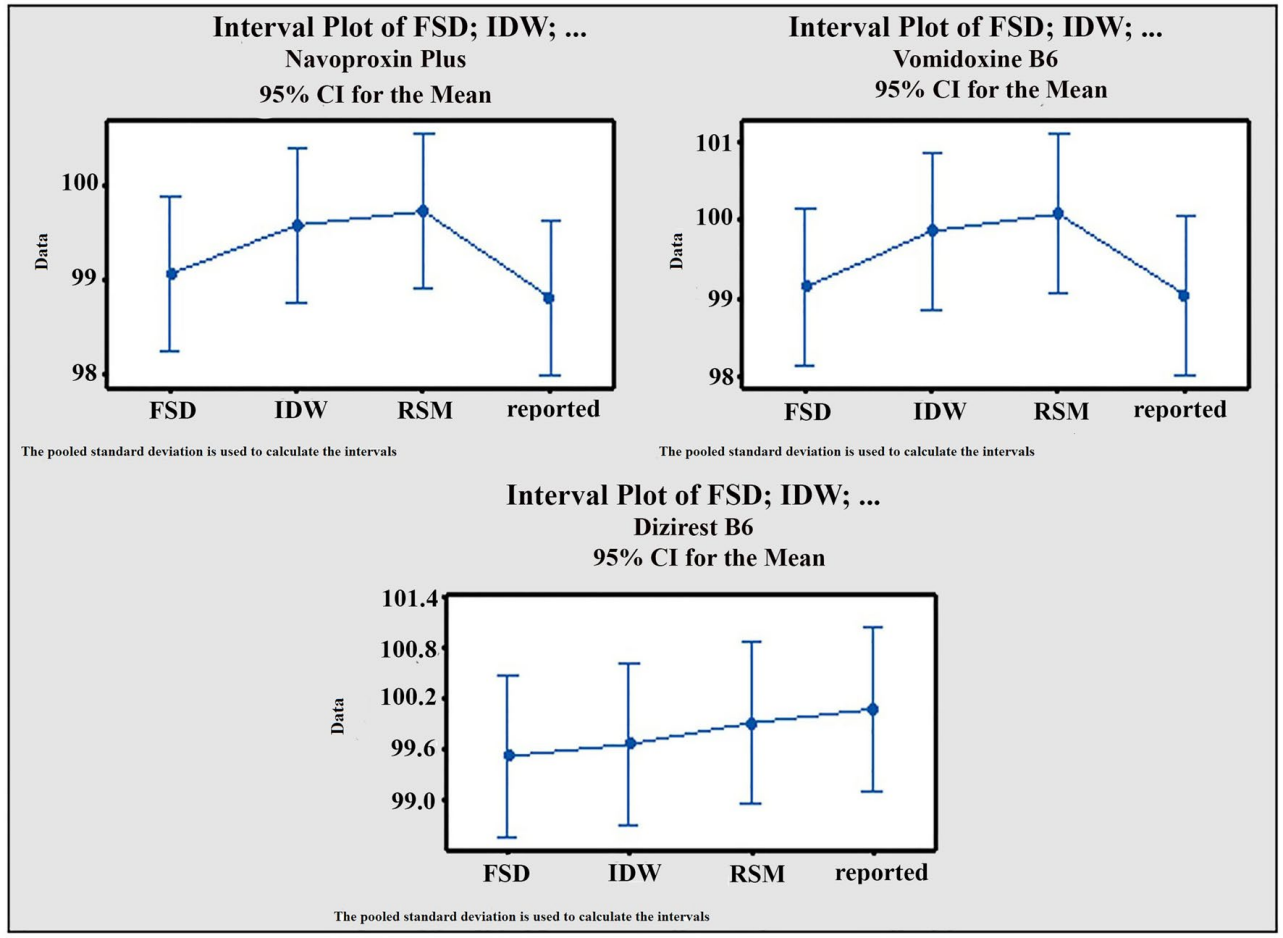
Interval plot for the proposed and reported methods of MZH in different pharmaceutical formulations

The second tool was the interval plot test [34]. Plots display confidence interval as vertical lines, with the center point corresponding to the interval mean. Assume that the data group intervals of each approach overlap each other in the diagram. These plots show that there is no considerable difference between offered and reported approaches in different pharmaceutical formulations, Fig. 7.

**Fig. 8:**
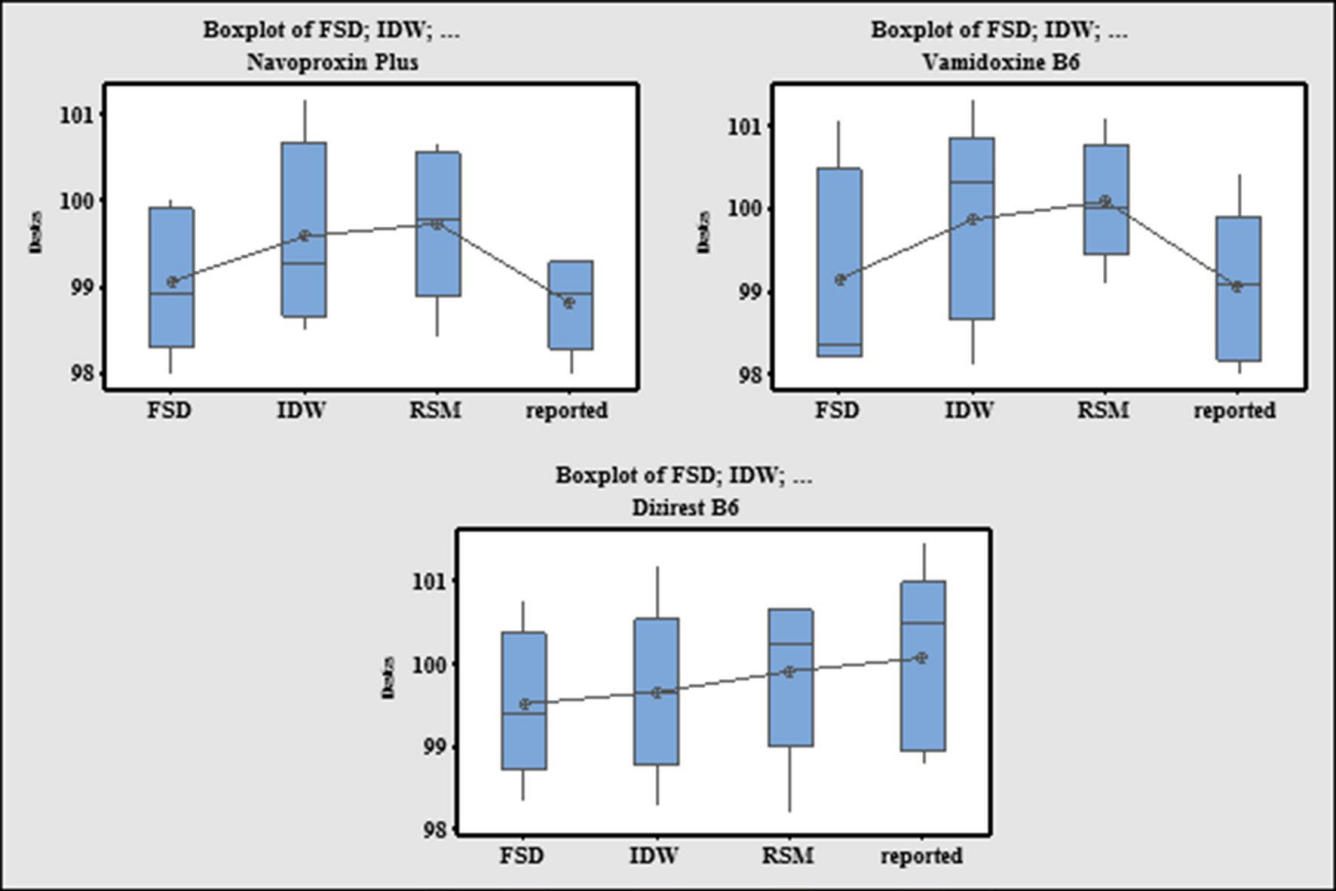
Box plot for the proposed and reported methods of MZH in different pharmaceutical formulations

The Boxplot is yet another important data visualization tool [34], which depicts the distribution of data between groups, Fig. 8 shows the proposed and reported approaches boxplots in different pharmaceutical formulations. The middle quartile is represented by the central box, which has a line in it that indicates the data median, upper lines that represent higher values, and whiskers that represent lower values. The distribution of data in each data category is depicted in the boxplot.

**Fig. 9:**
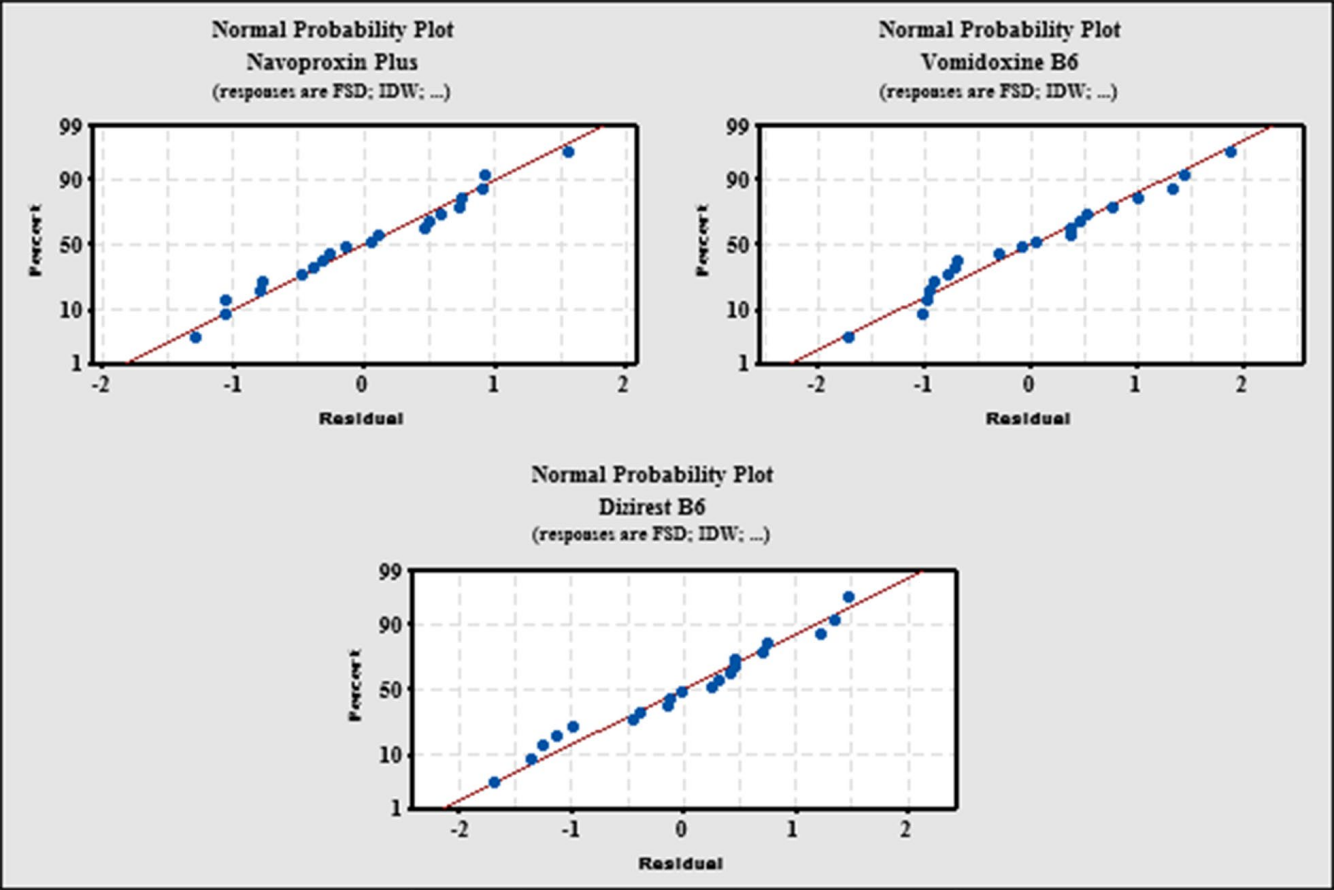
Normal probability plot for the proposed and reported methods of MZH in different pharmaceutical formulations

The normal probability plot [35] is another technique for determining if data is normally distributed Fig. 9. The normal distribution is satisfied in the data if the straight line goes through majority of the data points in different pharmaceutical formulations.

**Fig. 10:**
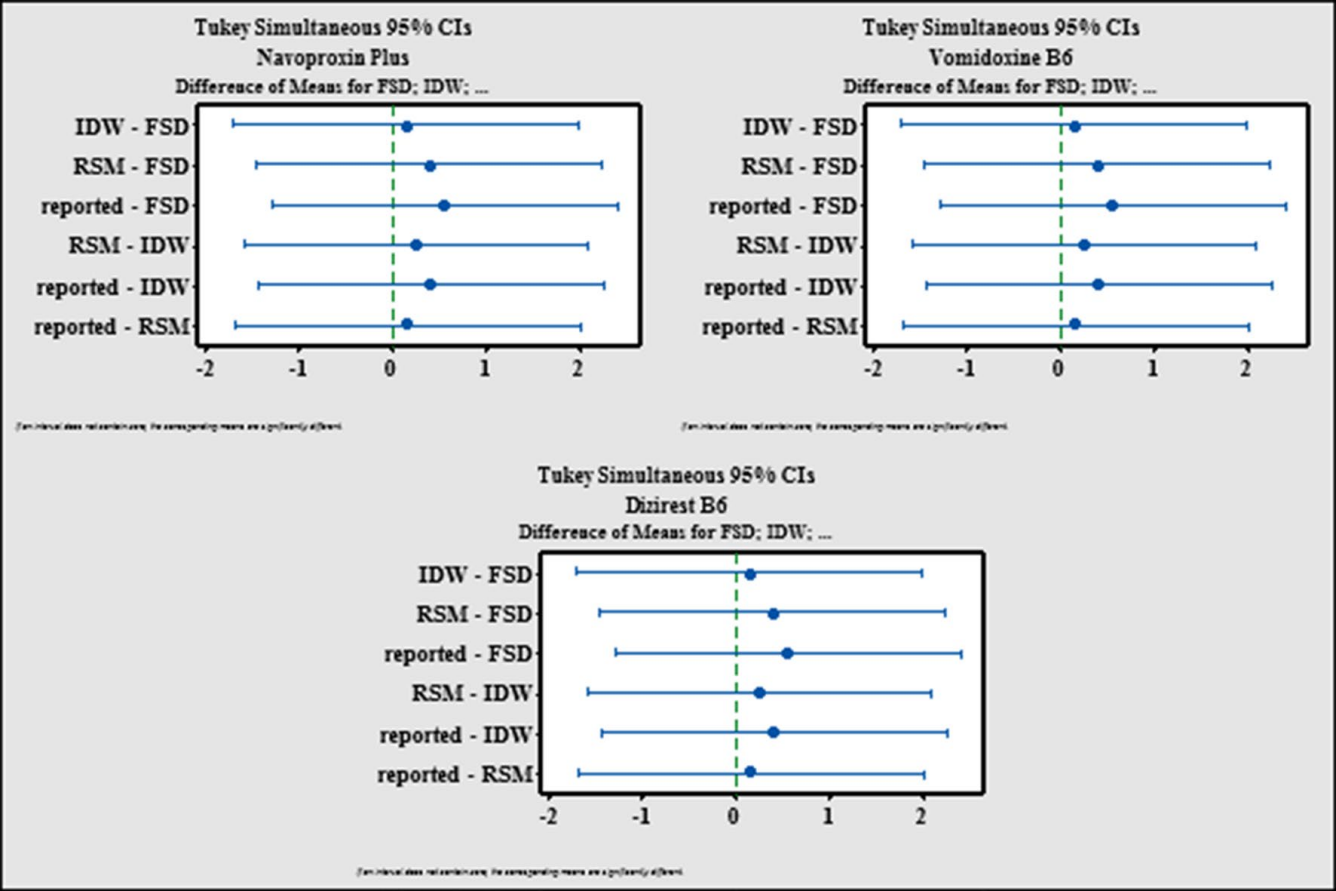
Tukey’s simultaneous significant difference test for the proposed and reported methods of MZH in different pharmaceutical formulations

Tukey’s simultaneous significant difference test [36] is the final statistical tool. It is a powerful tool for detecting any differences in the mean values of the distinct groups. Figure 10 depicts data interval for each group as a horizontal line with a central dot passing through the mean value of each data group. The overlap between the intervals suggested that the mean values of the proposed and reported approaches did not differ significantly in different pharmaceutical formulations.

### Comparison to reported methods

Both the suggested and reported procedures were analyzed side-by-side to determine whether one was more reliable (Table 8). Based on the data, it was determined that the RSM for MZH and Direct determination for PYH had the lowest LOD and lowest LOQ in comparison to the HPLC reported methods [17, 20].

**Table 8:** Comparison between proposed and reported methods of MZH and PYH

Methods	Proposed method	Proposed method	Proposed method	Proposed method	Reported method [17]	Reported method [20]
Techniques	RSM	IDW	FSD	Direct at 290 nm	HPLC–UV	HPLC–UV
Linear range (µg/mL)						
MZH	5–50	5–50	5–50		203–304	160–410
PYH				5–50	395–592	270–760
LOD (µg/mL)						
MZH	0.52	0.87	1.11		3.75	0.79
PYH				0.43	1.90	3.48
Application	Navoproxin plus^®^ tablets				Pyrimac^®^ tablets	Vominore^®^ tablets
	Vomidoxine B6^®^ tablets					
	Dizirest B6^®^ tablets					

## Conclusion

Three spectrophotometric techniques were used in this research to assess MZH in the presence of PYH in their pure powdered form, laboratory-prepared mixtures, and pharmaceutical formulations. These techniques benefit from being straightforward, involving just a few zero-order spectral mathematical calculations and a fundamental computing procedure. A statistical study utilizing the t-test and the F-test revealed no significant difference between the planned and stated spectrophotometric approaches. To help in data visualization, interval plots, boxplots, normal probability plots, Tukey’s simultaneous significant difference test, one-way ANOVA and two-way ANOVA were used to establish that there were no significant differences in the results of the proposed method with reported methods and with each other. The suggested methods have a very small impact on the environment because they meet all of NEMI's greenness criteria, GAPI, AGREE, and analytical Eco-scale.

## Data Availability

All data generated or analyzed during this study are included in this published article.
